# The protective role of MDL-1 in sepsis-induced lung injury: insights from a murine CLP model

**DOI:** 10.3389/fimmu.2026.1709000

**Published:** 2026-03-11

**Authors:** Baoji Hu, Yunpeng Wang, Daoqin Gao, Mengzhi Pan, Yangliang Yang, Yuan Yuan, Lulong Bo, Yi Zhang

**Affiliations:** 1 Department of Anesthesiology, Shanghai Pudong Hospital, Fudan University Pudong Medical Center, Shanghai, China; 2 School of Gongli Hospital Medical Technology, University of Shanghai for Science and Technology, Shanghai, China; 3 Department of Anesthesiology, Chinese People’s Liberation Army 92493 Hospital, Huludao, Liaoning, China; 4 Faculty of Anesthesiology, Changhai Hospital, Naval Medical University, Shanghai, China; 5 Department of Otorhinolaryngology Head and Neck Surgery, Pudong Gongli Hospital, Shanghai University of Medicine & Health Sciences, Shanghai, China

**Keywords:** inflammation, lung injury, MDL-1, murine, sepsis

## Abstract

**Introduction:**

The Myeloid DAP12-associating lectin-1 (MDL-1) serves as a pivotal pattern recognition receptor crucial for recognizing various pathogenic microorganisms and orchestrating immune responses during infections. This study aimed to elucidate the role of MDL-1 in the pathogenesis of sepsis-associated acute lung injury (ALI).

**Methods:**

Experiments were conducted using wild-type (WT) and MDL-1-deficient (MDL-1^-/-^) mice on a C57BL/6 background. Sepsis was induced by cecal ligation and puncture (CLP) surgery, with sham-operated mice serving as controls. Lung tissues were harvested 24 hours post-CLP for histopathological evaluation using hematoxylin and eosin (H&E) staining. MDL-1 expression on monocytes/macrophages was analyzed by flow cytometry. Cytokines of IFN-γ, IL-6, and TNF-α were measured via ELISA. Protein expression was assessed by western blot, and mRNA levels of key cytokines and chemokines in lung tissues were quantified using real-time PCR.

**Results:**

Survival analysis revealed that MDL-1 deficiency significantly exacerbated mortality in septic mice. In the CLP-induced sepsis model, the 7-day survival rate was 78.6% in the WT-CLP group, whereas it was markedly reduced to 42.9% in the MDL-1^-/-^-CLP group, demonstrating a statistically significant difference between the groups (P < 0.01). Histopathological assessment further confirmed that lung tissue damage was more severe in MDL-1^-/-^-CLP mice compared to their WT-CLP counterparts, as evidenced by a significantly higher composite injury score. MDL-1 expression was markedly upregulated on monocytes and macrophages (P < 0.01). Serum IL-6 and TNF-α levels were significantly elevated, whereas IFN-γ was reduced (P < 0.01). mRNA expression of cytokines and chemokines was correspondingly altered (P < 0.01). Furthermore, protein levels of phospho-Syk (p-Syk) and cleaved caspase-3 in lung tissues were significantly increased (P < 0.01).

**Discussion:**

Our findings demonstrate that MDL-1 deficiency exacerbates lung inflammation in a CLP-induced murine sepsis model. These results underscore the essential immunomodulatory role of MDL-1 in mitigating excessive pulmonary inflammatory responses during sepsis.

The Myeloid DAP12-associating lectin-1 (MDL-1) serves as a pivotal pattern recognition receptor crucial for recognizing various pathogenic microorganisms and orchestrating immune responses during infections. In this study, we elucidate that MDL-1 exhibits predominant expression within the mononuclear phagocyte system and plays a significant role in modulating the severity of lung injury in sepsis. Notably, MDL-1-deficient (MDL-1^-/-^) mice subjected to cecal ligation and puncture (CLP) exhibited elevated levels of pro-inflammatory cytokines such as IL-6 and TNF-α, along with decreased production of IFN-γ. Moreover, the expression levels of chemokines like IP-10, KC, MCP-1, and MIP-1 were markedly upregulated in septic MDL-1^-/-^ mice. Concurrently, enhanced levels of Syk and cleaved caspase 3 were detected in the lung tissues of MDL-1^-/--^CLP animals, underscoring an augmented inflammatory response. Collectively, our findings indicate that MDL-1 deficiency correlates with the exacerbation of lung inflammation in a murine model of CLP-induced sepsis, highlighting the critical immunomodulatory role of MDL-1 in safeguarding against excessive pulmonary inflammation.

## 1 Introduction

Sepsis is a critical medical condition characterized by life-threatening organ dysfunction resulting from a dysregulated host response to infection, presenting a formidable challenge in clinical practice. The etiology of sepsis can vary based on geographical factors and patient demographics, with bacterial infections predominantly affecting the respiratory and gastrointestinal systems (1). Lung impairment during sepsis manifests as an acute, widespread inflammatory pulmonary injury characterized by heightened alveolar capillary permeability, interstitial and alveolar edema, and loss of aerated lung parenchyma. A myriad of immune cells, particularly macrophages and monocytes, are intricately involved in this pathological cascade through the secretion of chemokines and cytokines, thus amplifying the inflammatory milieu (2). Activation of the mononuclear phagocyte system can further exacerbate tissue damage, potentially inducing epithelial cell apoptosis via IFN-β-mediated release of tumor necrosis factor (TNF)-related apoptosis-inducing ligand (TRAIL), which engages death receptors and aggravates lung injury (3).

Central to our investigation is Myeloid DAP12-associating lectin-1 (MDL-1), also recognized as myeloid C-type lectin 5A (CLEC5A). MDL-1 is a receptor intricately linked with spleen tyrosine kinase (Syk) signaling and is prominently expressed on monocytes, macrophages, and neutrophils. MDL-1 expression is augmented by interferon-gamma (IFN-γ), positioning it at a vital nexus of immune regulation (4, 5). Prior studies have revealed that MDL-1 activation by arboviruses such as dengue and Japanese encephalitis virus leads to the release of key pro-inflammatory mediators like tumor necrosis factor-alpha (TNF-α), interleukin-6 (IL-6), KC (CXCL1), and interferon-inducible protein (IP)-10, underscoring MDL-1’s pivotal role in immune responses (5, 6). Additionally, the involvement of MDL-1 in diverse inflammatory conditions, such as collagen-induced rheumatoid arthritis and Concanavalin A-induced liver inflammation, highlights its multifaceted immunomodulatory functions (4, 7). Recent findings on MDL-1’s interaction with exosomes released from activated platelets further accentuate its role in mediating inflammation (8). Despite its established association with inflammatory processes, the specific involvement of MDL-1 in sepsis-induced lung injury remains unexplored.

Herein, our study elucidates that MDL-1 deficiency exacerbates lung injury in the setting of sepsis. Beyond its role in cytokine production, MDL-1 emerges as a pivotal regulator in orchestrating immune cell recruitment within lung tissues. Noteworthy findings include the upregulation of Syk and cleaved caspase 3 expression in MDL-1-deficient mice, which are indicative of apoptotic processes. Moreover, the increased mortality rate observed in MDL-1^-/-^ mice following CLP underscores the detrimental consequences of MDL-1 deficiency in exacerbating lung inflammation. In summation, our data suggest that a deficiency in MDL-1 could foster increased lung injury and inflammation in a murine CLP model, shedding new light on the intricate interplay between MDL-1 and sepsis-related pulmonary pathology.

## 2 Materials and methods

### 2.1 Experimental animals

C57BL/6 mice were obtained from the Research Animal Center of Shanghai Pudong Hospital (Shanghai, China). MDL-1-deficient mice (MDL-1^-/-^) on a C57BL/6 background were procured from Shanghai Model Organisms (Contract No. 2018-W-1550 N1-2491) and utilized in this study. All animals were maintained in a specific pathogen-free (SPF) environment under controlled conditions, including a 12-hour light/dark cycle, ambient temperature of 22 ± 2 °C, and relative humidity of 50 ± 10%. Standard laboratory chow and autoclaved water were provided ad libitum.

To ensure the health and welfare of the animals, regular veterinary monitoring was conducted, and all mice were acclimatized for at least one week prior to experimentation. All experimental procedures were approved by the Institutional Animal Care and Use Committee (IACUC) of Shanghai Pudong Hospital and conducted in strict compliance with the Guide for the Care and Use of Laboratory Animals (National Institutes of Health, 8th Edition) and relevant ethical guidelines and regulations. Efforts were made to minimize animal suffering and reduce the number of animals used, adhering to the principles of the 3Rs (Replacement, Reduction, and Refinement).

### 2.2 Experimental design and CLP model of sepsis

Mice were stratified into four distinct groups based on experimental objectives (1): the wild-type (WT) mice subjected to sham surgery (control) group (2), WT mice subjected to CLP-induced sepsis ((WT-)CLP) group (3), MDL-1-deficient mice subjected to sham surgery (MDL-1^-/-^) group, and (4) MDL-1-deficient mice subjected to CLP-induced sepsis (MDL-1^-/--^CLP) group.

Sepsis was induced using the CLP method, as previously described (9). Briefly, mice were anesthetized with sevoflurane, and a midline abdominal incision was made to expose the cecum. The cecum was ligated at a defined point and punctured once with a 21-gauge needle to induce polymicrobial peritonitis. The cecum was gently squeezed to extrude a small amount of fecal content into the peritoneal cavity, ensuring consistent severity of sepsis across all animals. The abdominal wall was then closed in layers using sterile sutures, and mice were resuscitated with a subcutaneous injection of 0.9% sodium chloride solution (1 mL per mouse) to prevent dehydration and shock. In the sham group, mice underwent laparotomy without cecal ligation or puncture to serve as surgical controls.

Postoperative care included monitoring for signs of distress, providing analgesia, and maintaining a warm environment to prevent hypothermia. The survival rate of both C57BL/6 and MDL-1^-/-^ mice was monitored for 7 days following CLP. Tissue and blood samples were collected 24 hours post-operation for subsequent analyses.

### 2.3 Hematoxylin-eosin staining

To evaluate the effect of MDL-1 on lung injury, lung tissues were harvested and fixed in 10% neutral buffered formalin for 24 hours. Following fixation, tissues were dehydrated in a graded ethanol series, cleared in xylene, and embedded in paraffin blocks. Sections were cut at a thickness of 5 μm using a microtome and mounted onto glass slides.

For histological analysis, sections were deparaffinized, rehydrated, and stained with hematoxylin and eosin (H&E) following standard protocols. Hematoxylin stained cell nuclei blue, while eosin counterstained cytoplasmic and extracellular matrix components pink, enabling clear visualization of tissue morphology.

Microscopic evaluation was performed by two blinded investigators using a light microscope at 100×, 200×, 400× and 600× magnification. Lung injury was assessed based on the following pathological features (1): alveolar congestion (2), hemorrhage (3), inflammatory cell infiltration, and (4) alveolar wall thickness. A semi-quantitative scoring system was employed to grade the severity of lung injury, with total scores ranging from 0 (no injury) to 4 (severe injury). The total lung injury score was calculated as the sum of individual scores for all four parameters.

Representative images were captured using a digital imaging system, and quantitative data were analyzed to compare lung injury severity between experimental groups.

### 2.4 Flow cytometry analysis

To investigate the expression of MDL-1 in lung and blood cells during sepsis, a comprehensive flow cytometry protocol was employed.

Lung tissues were dissected and cut into small pieces (1–2 mm in size) using sterile surgical instruments. Tissue fragments were washed three times with ice-cold phosphate-buffered saline (PBS) to remove residual blood and debris. Subsequently, the samples were enzymatically digested by incubating with 0.15% trypsin solution at 37 °C for 15 minutes under constant shaking to dissociate cells. The digested tissue suspension was filtered through a 300-mesh (50 μm, Beyotime Biotechnology, Shanghai, China; Catalog No. 431002) nylon sieve to remove undigested clumps and obtain a single-cell suspension. The filtrate was washed twice with PBS and centrifuged at 300 × g for 5 minutes to pellet the cells.

Blood samples were collected via cardiac puncture using a heparinized syringe to prevent coagulation. The blood was immediately transferred to sterile tubes and processed for flow cytometry analysis.

The single-cell suspension was stained with fluorochrome-conjugated antibodies specific for CD11b (APC, clone, M1/70, eBioscience Shanghai, China), CD11c (BUV395, clone, HL3, BD Biosciences Shanghai, China), Siglec-F(BV650, clone, E50-2440, BD Biosciences Shanghai, China), CD45(Alexa Fluor™ 488, clone, I3/2.3, BD Biosciences Shanghai, China), CD115 (PE, clone, AFS98, eBioscience Shanghai, China), F4/80 (PE, clone, T45-2342, BD Biosciences Shanghai, China), MDL-1 (FITC, clone, 120504, eBioscience Shanghai, China) and isotype control (for MDL-1) (FITC, clone, Rat IgG2b, κ, eBioscience Shanghai, China). Staining was performed in the dark at 4 °C for 30 minutes, followed by two washes with PBS to remove unbound antibodies.

Flow cytometry was conducted using a BD FACSCanto II flow cytometer (BD Biosciences, USA). Data acquisition and analysis were performed using FACSDiva software (BD Biosciences). Gating strategies and PMT voltages used for flow cytometry analysis. All gating was performed using FACSDiva software (BD Biosciences), with single-cell gating confirmed via FSC-A/FSC-H plots and isotype controls used to define MDL-1-positive thresholds, and the expression levels of MDL-1 were quantified as mean fluorescence intensity (MFI).

### 2.5 Enzyme-linked immunosorbent assay

To quantify the expression of key chemokines and cytokines in lung tissues, an enzyme-linked immunosorbent assay (ELISA) was performed.

Lung tissues were carefully dissected and placed in ice-cold phosphate-buffered saline (PBS, pH 7.0) supplemented with protease inhibitors to prevent protein degradation. The PBS buffer contained the following components: 0.002% sodium azide (to inhibit microbial growth), 0.1 mg/mL soybean trypsin inhibitor (to block trypsin activity), 2 mM phenylmethylsulfonyl fluoride (PMSF, a serine protease inhibitor), 10 nM ethylenediaminetetraacetic acid (EDTA, a metalloprotease inhibitor), and 1.0 mg/mL bovine serum albumin (BSA, to stabilize proteins). Tissues were homogenized on ice using a tissue homogenizer to ensure complete cell lysis.

The homogenized samples were incubated at 4 °C for 2 hours to allow for complete protein extraction. Following incubation, samples were centrifuged at 12, 000 × g for 10 minutes at 4 °C to separate cellular debris. The resulting supernatants were carefully collected and stored at −80 °C until further analysis.

Cytokine levels, including IFN-γ, IL-6, and TNF-α, were quantified in the supernatants using commercially available ELISA kits (Invitrogen, Carlsbad, CA, USA). The assays were performed according to the manufacturer’s instructions. Briefly, 96-well plates pre-coated with capture antibodies specific to each cytokine were incubated with supernatants and standard solutions. After washing to remove unbound proteins, detection antibodies conjugated to horseradish peroxidase (HRP) were added. Tetramethylbenzidine (TMB) substrate was used for color development, and the reaction was stopped with 2 N sulfuric acid. Absorbance was measured at 450 nm using a microplate reader (BioTek Instruments, Winooski, VT, USA).

Cytokine concentrations were determined by interpolating absorbance values against a standard curve generated using known concentrations of recombinant cytokines. All samples were assayed in duplicate, and results were expressed as picograms per milliliter (pg/mL) of supernatant.

### 2.6 Real-time PCR

To evaluate the mRNA expression levels of key cytokines and chemokines in lung tissues, a quantitative real-time PCR (qPCR) assay was performed.

Total RNA was extracted from lung tissues using TRIzol reagent (Takara Biotechnology, Dalian, China) according to the manufacturer’s protocol. Briefly, tissues were homogenized in TRIzol, and RNA was isolated by phase separation with chloroform, followed by precipitation with isopropanol. The RNA pellet was washed with 75% ethanol, air-dried, and resuspended in RNase-free water. RNA concentration and purity were determined using a NanoDrop spectrophotometer (Thermo Fisher Scientific, Waltham, MA, USA), with A260/A280 ratios between 1.8 and 2.0 considered acceptable.

Reverse transcription was performed using a PrimeScript RT reagent kit (Takara Biotechnology, Dalian, China). For each reaction, 1 µg of total RNA was reverse-transcribed into complementary DNA (cDNA) in a 20 µL reaction volume containing oligo(dT) primers, random hexamers, and reverse transcriptase. The reaction was carried out at 37 °C for 15 minutes, followed by 85 °C for 5 seconds to inactivate the enzyme.

Gene-specific primers for cytokines and chemokines including: IFN-γ, IL-6, TNF-α, IP-10, KC, Monocyte Chemotactic Protein 1 (MCP-1), Monokine Induced by Interferon-γ (MIG), Macrophage Inflammatory Protein 1(MIP-1) and β-actin (internal control) were designed using Primer-BLAST (NCBI) and synthesized by a commercial provider. The sequences of the primers used in this study are demonstrated in **Table 1**.

**Table 1:** The sequences of the primers.

Primers	Sense (5′-3′)	Antisense (5′-3′)
IFN-γ	GGTCATTCAGATGTAGCGG	CACTCTCCTCTTTCCAATTC
IL-6	TACCACTCCCAACAGACCTG	GGTACTCCAGAAGACCAGAGG
TNF-α	AATGGCCTCCCTCTCATCAG	CCCTTGAAGAGAACCTGGGA
IP-10	AATGCCACCCATTGCCAGTA	TAGCTCGAAAACGCCTCTGG
KC	CAATGAGCTGCGCTGTCAGTG	CTTGGGGACACCTTTTAGCATC
MCP-1	GTGTAAGAGGCTGGGAGTGC	CTGACTCTGCGGAAGTCTCC
MIG	TGCTAGAGGCAAAAACTCTGTG	TAGGCTCAAGGGCGTGAT
MIP-1	CAGCTTATAGGAGATGGAGCTATG	TCACTGACCTGGAACTGAATG
β-actin	GGCTGTATTCCCCTCCATCG	CCAGTTGGTAACAATGCCATGT

qPCR was performed using the SYBR Green PCR Master Mix (Takara Biotechnology, Dalian, China) in an ABI 7500 Real-Time PCR System (Applied Biosystems, Foster City, CA, USA). Each reaction (20 µL) contained 10 µL of SYBR Green Master Mix, 1 µL of cDNA template, and 0.5 µM of each primer. The thermal cycling conditions were as follows: initial denaturation at 95 °C for 3 minutes, followed by 40 cycles of denaturation at 95 °C for 10 seconds, annealing at 60 °C for 5 seconds, and extension at 72 °C for 10 seconds. A melt curve analysis was performed at the end of the reaction to confirm the specificity of amplification.

The relative mRNA expression levels of target genes were normalized to the housekeeping gene β-actin using the 2−ΔΔCt method. All samples were assayed in triplicate, and results were expressed as fold changes relative to the control group.

### 2.7 Western blot analysis

To investigate the molecular mechanisms underlying MDL-1’s role in sepsis-induced lung injury, immunoblotting was performed to assess the expression of key proteins involved in apoptotic and signaling pathways.

Lung tissues were homogenized in ice-cold RIPA lysis buffer (50 mM Tris-HCl pH 8.0, 150 mM NaCl, 1 mM EDTA pH 8.0, 2% SDS, 1% NP-40, and 5 mM DTT) supplemented with protease and phosphatase inhibitors to prevent protein degradation. The homogenates were centrifuged at 12, 000 × g for 15 minutes at 4 °C, and the supernatants were collected. Protein concentrations were quantified using a bicinchoninic acid (BCA) assay kit (Beyotime, Shanghai, China) according to the manufacturer’s instructions.

Equal amounts of protein (30 µg per lane) were resolved on 10% sodium dodecyl sulfate-polyacrylamide gel electrophoresis (SDS-PAGE) under reducing conditions. The separated proteins were then electrophoretically transferred onto polyvinylidene difluoride (PVDF) membranes (Immobilon, Merck KGaA, Darmstadt, Germany) using a wet transfer system. Transfer efficiency was confirmed by staining the membranes with Ponceau S solution.

Membranes were blocked with 5% non-fat dry milk in Tris-buffered saline containing 0.1% Tween-20 (TBST) for 1 hour at room temperature to prevent non-specific binding. Subsequently, the membranes were incubated with the following primary antibodies diluted in blocking buffer:

Cleaved Caspase-3 (Cell Signaling Technology, Beverly, MA, USA; 1:1000).

Syk (Cell Signaling Technology, Beverly, MA, USA; 1:1000).

β-actin (Cell Signaling Technology, Beverly, MA, USA; 1:1000, used as an internal control).

Primary antibody incubation was performed at 4 °C for 4 hours or overnight with gentle agitation.

After washing three times with TBST, the membranes were incubated with horseradish peroxidase (HRP)-conjugated secondary antibodies (Cell Signaling Technology, Beverly, MA, USA; 1:2000) for 1 hour at room temperature. Protein bands were visualized using an enhanced chemiluminescence (ECL) detection kit (Pierce, Rockford, IL, USA) according to the manufacturer’s instructions. Chemiluminescent signals were captured using a ChemiDoc Imaging System (Bio-Rad Laboratories, Hercules, CA, USA).

The optical density (OD) of protein bands was quantified using ImageJ software (National Institutes of Health, Bethesda, MD, USA). The expression levels of target proteins were normalized to β-actin to account for variations in protein loading. Results were expressed as fold changes relative to the control group.

The expression of cleaved caspase-3 was used as a marker of apoptosis, while Syk expression was assessed to evaluate its role in MDL-1-mediated signaling pathways. These findings provide insights into the molecular mechanisms by which MDL-1 modulates sepsis-induced lung injury.

### 2.8 Statistical analysis

Statistical analyses were performed to evaluate the significance of experimental findings and ensure robust interpretation of the data. All analyses were conducted using GraphPad Prism 9 (GraphPad Software Inc., San Diego, CA, USA), a widely used statistical software for biological and medical research.

Continuous data were expressed as mean ± standard error of the mean (SEM) to account for variability within the experimental groups. SEM was chosen as it provides a measure of the precision of the sample mean and is particularly useful for comparing group means in biological studies.

To compare the means of three or more independent groups, one-way Analysis of Variance (ANOVA) was employed. This test was chosen because it is appropriate for assessing differences in continuous variables across multiple experimental conditions. *Post hoc* analysis was performed using Tukey’s multiple comparisons test to identify specific group differences while controlling for Type I error.

For survival analysis, the log-rank test was used to compare survival curves between groups. This non-parametric test is widely used in time-to-event studies and is particularly suited for assessing differences in survival outcomes in sepsis-induced lung injury models.

A p-value < 0.05 was considered statistically significant, indicating that the observed differences were unlikely to have occurred by chance. All statistical tests were two-tailed unless otherwise specified.

Prior to analysis, data were assessed for normality using the Shapiro-Wilk test and for homogeneity of variance using Bartlett’s test. If assumptions of normality or equal variance were violated, appropriate non-parametric tests (e.g., Kruskal-Wallis test) were applied. Outliers were identified using the Grubbs’ test and excluded if they significantly skewed the results.

To ensure the study had sufficient statistical power, a power analysis was conducted using preliminary data. The sample size was determined to achieve a power of 80% with an alpha level of 0.05, ensuring the ability to detect biologically relevant differences.

## 3 Results

### 3.1 MDL-1 deficiency exacerbates mortality in septic mice

Our study revealed a profound and distinct impact of MDL-1 deficiency on the survival outcomes of mice subjected to CLP-induced sepsis. The survival rates were meticulously monitored over a 7-day period to evaluate the role of MDL-1 in conferring protection against sepsis-induced mortality.

Notably, MDL-1^-/-^ mice subjected to sham surgery exhibited 92.9% survival over 7 days observation period. There is no difference with the WT - sham group (100% survival), indicating that MDL-1 deficiency alone does not affect baseline survival in the absence of sepsis induction.

**Figure 1:**
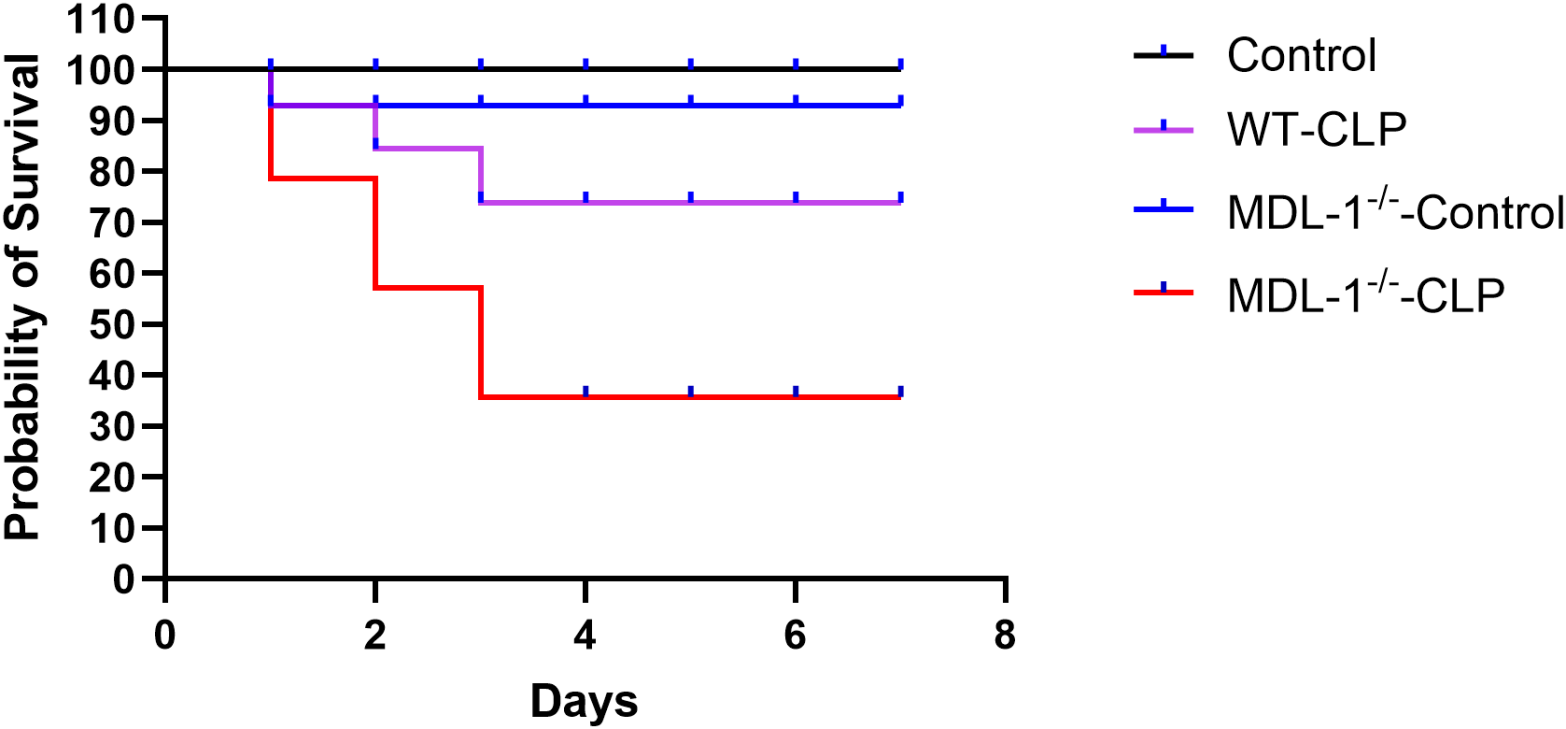
Survival rates of wild-type (WT) and MDL-1-deficient (MDL-1^-/-^) mice subjected to CLP-induced sepsis or sham surgery over 7 days. Survival rates: control (100%), WT-CLP (78.6%), MDL-1^-/-^-control (92.9%), MDL-1^-/-^ -CLP (42.9%). n=14 per group. Statistical significance was determined by log-rank (Mantel-Cox) test (P < 0.01, MDL-1^-/-^ - CLP vs. WT - CLP; no significance between sham groups).

In the WT-CLP group, the 7-day survival rate was 78.6% (11 out of 14), indicating that wild-type mice exhibited a relatively robust survival response to sepsis. In contrast, the MDL-1^-/-^ -CLP group demonstrated a significant decline in survival, with only 42.9% (6 out of 14) of mice surviving the 7-day observation period (**Figure 1**). Comparative statistical analysis using the log-rank test revealed a significant difference in survival rates between the MDL-1^-/-^ -CLP and WT-CLP groups (P < 0.01). This striking disparity highlights the critical role of MDL-1 in mitigating the lethal consequences of sepsis. The heightened susceptibility to mortality observed in MDL-1^-/-^ mice underscores the detrimental impact of MDL-1 deficiency in the context of CLP-induced sepsis. These findings suggest that MDL-1 plays a protective role in enhancing survival during systemic inflammatory responses, potentially through its involvement in modulating immune and cellular pathways. This study provides compelling evidence that MDL-1 deficiency exacerbates sepsis-induced mortality, emphasizing the importance of MDL-1 as a potential therapeutic target for improving survival outcomes in sepsis.

### 3.2 MDL-1 deficiency exacerbates lung injuries in septic mice

To evaluate the impact of MDL-1 deficiency on sepsis-induced lung injury, lung specimens were collected 24 hours post-CLP induction and subjected to hematoxylin and eosin (H&E) staining for histopathological analysis. This time point was chosen to capture the acute phase of lung injury, which is critical for understanding the early pathological changes in sepsis.

**Figure 2:**
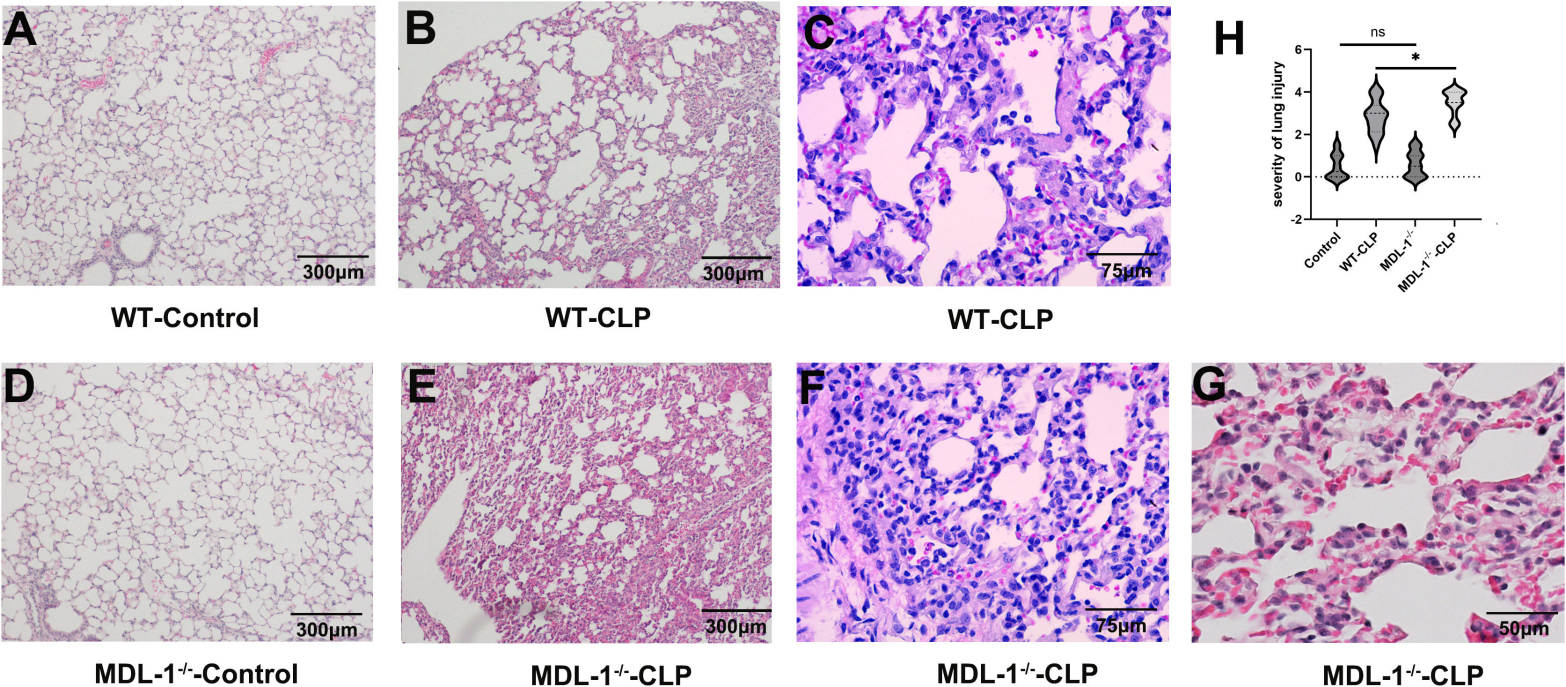
Histopathological analysis of lung injury in WT and MDL-1^-/-^ mice. Lung specimens were collected 24 hours post-CLP induction and subjected to hematoxylin and eosin **(H, E)** staining for histopathological analysis. Representative H&E-stained sections of WT lung tissues from **(A)** sham group (WT-control, × 100 magnification), CLP group **(B)** (WT-CLP, × 100 magnification) and **(C)** (WT-CLP, × 400 magnification). MDL-1^-/-^ lung tissues from **(D)** sham group (MDL-1^-/-^-control, × 100 magnification), CLP group **(E)** (MDL-1^-/-^-CLP, × 100 magnification), **(F)** (MDL-1^-/-^-CLP, × 400 magnification) and **(G)** (MDL-1^-/-^-CLP, × 600 magnification). **(H)** The violin plot of semi-quantitative histopathological scores of lung injury. Histopathological analysis of lung injury in WT and MDL-1^-/-^ mice. Results are depicted as the mean ± SEM. (*p<0.05, n=16/group, ns, no significance between sham groups) Kruskal-Wallis test.

Notably, the MDL-1^-/-^ control group (sham surgery) showed no significant lung injury or excessive immune cell infiltration compared to WT controls (**Figure 2H**). This confirms that MDL-1 deletion alone does not induce baseline pulmonary inflammation, and the exacerbated immune cell infiltration and tissue damage observed in MDL-1^-/-^-CLP mice are specifically associated with sepsis induction rather than intrinsic effects of MDL-1 deficiency. As illustrated in **Figures 2A–G**, both the WT-CLP and MDL-1^-/-^ -CLP groups exhibited hallmark features of sepsis-induced lung injury, including interstitial edema, characterized by fluid accumulation in the lung interstitium. Alveolar hemorrhage, indicative of vascular damage and red blood cell leakage into alveolar spaces. Thickening of the pulmonary septum, reflecting structural alterations and inflammation. Infiltration of inflammatory cells, suggesting a robust immune response to systemic infection. Despite these shared features, the histopathological scores revealed a significant escalation in the severity of lung injury in the MDL-1^-/-^ -CLP group compared to the WT-CLP group (**Figure 2H**). While the absolute difference in total scores is modest, this finding, combined with complementary data on inflammatory cytokines, chemokines, and apoptotic markers (Sections 3.4–3.6), collectively supports the notion that MDL-1 deficiency contributes to enhanced lung tissue damage during sepsis.

The exacerbated lung pathology observed in MDL-1^-/-^ mice underscores the protective role of MDL-1 in mitigating tissue damage under septic conditions. The absence of MDL-1 appears to amplify the inflammatory response and structural damage in the lungs, suggesting that MDL-1 plays a critical role in regulating immune and cellular pathways involved in lung protection. These findings provide compelling evidence that MDL-1 deficiency worsens sepsis-induced lung injury, emphasizing its potential as a therapeutic target for reducing organ damage in sepsis. Further studies are warranted to elucidate the specific mechanisms by which MDL-1 modulates lung inflammation and tissue repair.

### 3.3 Wide distribution of MDL-1 on monocytes/macrophages

Monocytes and macrophages are pivotal players in the innate immune response, serving as the first line of defense against pathogenic threats and playing a central role in the progression of sepsis. To elucidate the involvement of MDL-1 in these critical immune cells, we investigated its expression patterns in both monocytes and macrophages under septic conditions.

**Figure 3:**
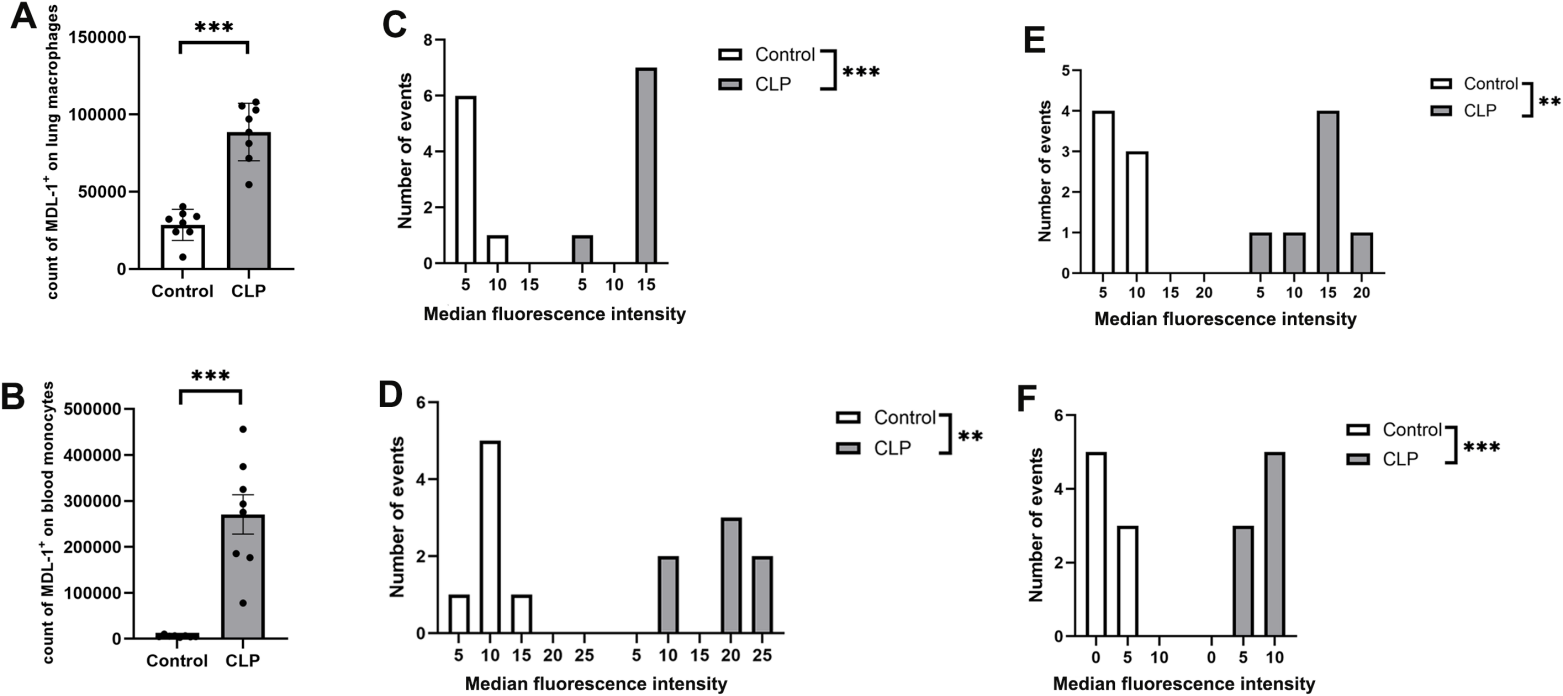
Flow cytometry analysis of MDL-1 surface expression. **(A)** Count of CD115^+^CD11b^+^ MDL-1^+^lung cells and **(B)** F4/80^+^CD11b^+^ MDL-1^+^ blood cells 24 hours after CLP (n=8 per group). The histograms of median fluorescence intensity (MFI) of MDL-1 on **(C)** CD115^+^CD11b^+^ lung macrophages, **(D)** F4/80^+^CD11b^+^ blood monocytes, **(E)** CD45^+^CD11c^+^Siglec-F^+^ alveolar macrophages, and **(F)** CD45^+^CD11c^-^CD11b^+^F4/80^+^ interstitial macrophages. Results are depicted as mean ± SEM. (n=8 per group **p < 0.01, ***P < 0.001; two-way ANOVA with Tukey’s post-test).

Our analysis revealed several key findings (1): The count of CD115^+^CD11b^+^ lung macrophages was significantly increased in WT-CLP mice compared to the sham group (P < 0.01; **Figure 3A**), indicating enhanced immune cell infiltration during sepsis (2). Critically, the median fluorescence intensity (MFI) of MDL-1 on these lung macrophages was substantially upregulated in WT-CLP mice (P < 0.01; **Figure 3C**), confirming increased MDL-1 expression per cell independent of cell number (3). A similar pattern was observed in blood F4/80^+^CD11b^+^ monocytes: WT-CLP mice exhibited both higher cell counts (P < 0.01; **Figure 3B**) and elevated MDL-1 MFI (P < 0.01; **Figure 3D**) compared to the sham group (4). Subset-specific flow cytometry analysis demonstrated that MDL-1 expression is upregulated in both major lung macrophage subsets: alveolar macrophages (CD45^+^CD11c^+^Siglec-F^+^) and interstitial macrophages (CD45^+^CD11c^-^CD11b^+^F4/80^+^). For alveolar macrophages, WT-CLP mice exhibited a significantly higher MDL-1 MFI compared to the sham group (P < 0.01; **Figure 3E**), while interstitial macrophages from WT-CLP mice also showed elevated MDL-1 MFI (P < 0.01; **Figure 3F**). These data collectively demonstrate that sepsis induces increased recruitment of monocyte/macrophage lineage cells and enhanced MDL-1 expression in both alveolar and interstitial macrophages—key subsets involved in pathogen recognition, inflammatory modulation, and tissue repair during sepsis.

These findings collectively emphasize the potential of MDL-1 as a key regulator of monocyte and macrophage activity in the pathogenesis of sepsis-induced lung injury. Understanding the mechanisms underlying MDL-1’s role in these cells could pave the way for novel therapeutic strategies aimed at enhancing immune responses and mitigating organ damage in sepsis.

### 3.4 Impact of MDL-1 deficiency on proinflammatory cytokine production

To further elucidate the role of MDL-1 in sepsis-induced lung injury, we investigated its influence on the production of key proinflammatory cytokines, including IFN-γ, IL-6, and TNF-α. These cytokines are critical mediators of the inflammatory response and play a pivotal role in the pathogenesis of sepsis-related organ damage.

**Figure 4:**
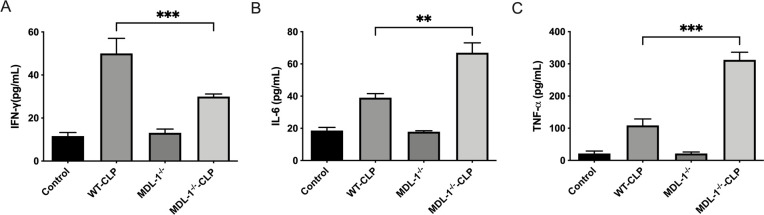
Serum cytokine levels in WT and MDL-1^-/-^ mice post-CLP, measured by ELISA. Serum cytokine levels in WT and MDL-1^-/-^ mice post-CLP, measured by ELISA. **(A)** IFN-γ, **(B)** TNF-α, **(C)** IL-6. Data are represented as mean ± SEM (n=5-8/group; **p < 0.01, ***p < 0.001), two-way ANOVA.

Our analysis revealed a significant increase in the levels of IFN-γ, (**Figure 4A**) IL-6 (**Figure 4B**), and TNF-α (**Figure 4C**) in the lung tissues of both WT-CLP and MDL-1^-/-^ -CLP mice compared to the control (sham) group (P < 0.01; **Figure 4**). This observation highlights the robust activation of the inflammatory response in the lungs during sepsis. Notably, the levels of IL-6 and TNF-α were substantially higher in the MDL-1^-/-^ -CLP group compared to the WT-CLP group (P < 0.01; **Figures 4B, C**). This suggests that MDL-1 deficiency exacerbates the inflammatory response, leading to an amplified release of these proinflammatory cytokines. Intriguingly, the level of IFN-γ was significantly lower in the MDL-1^-/-^ -CLP group compared to the WT-CLP group (P < 0.01; **Figure 4A**). This finding points to a unique regulatory role of MDL-1 in the production of IFN-γ, which may reflect its involvement in modulating specific immune pathways during sepsis.

The exacerbated production of IL-6 and TNF-α in MDL-1-deficient mice underscores the protective role of MDL-1 in tempering the inflammatory response. Conversely, the reduced levels of IFN-γ suggest that MDL-1 may also play a distinct role in regulating cytokine balance, potentially influencing the polarization of immune responses during sepsis.

These findings collectively highlight the critical involvement of MDL-1 in modulating the production of proinflammatory cytokines, thereby influencing the severity of lung injury in sepsis. The observed dysregulation of cytokine levels in MDL-1-deficient mice emphasizes the potential of MDL-1 as a therapeutic target for mitigating excessive inflammation and improving outcomes in sepsis.

### 3.5 Influence of MDL-1 absence on proinflammatory cytokine gene expression

**Figure 5:**
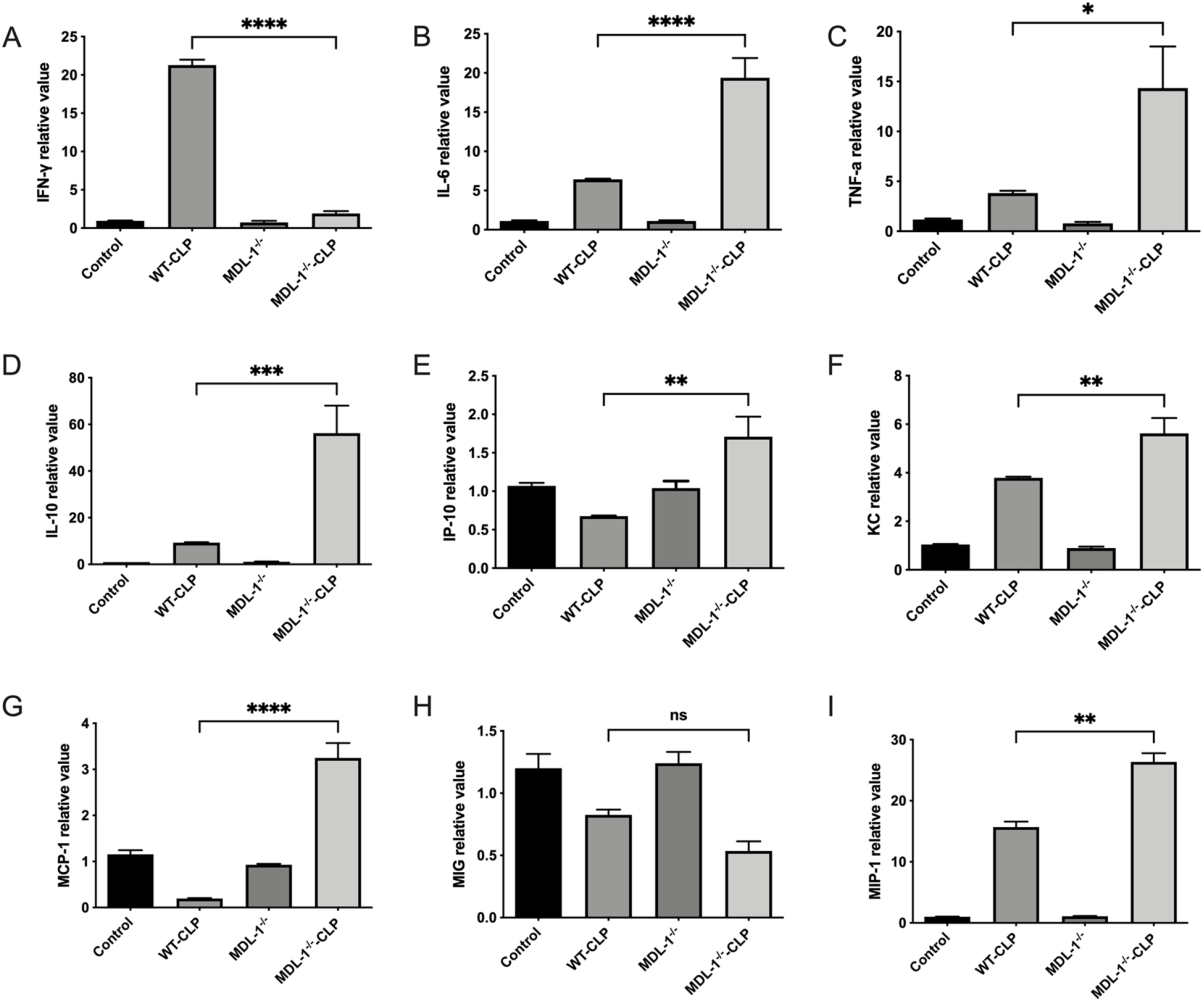
qRT-PCR analysis of cytokine/chemokine mRNA in lung tissues from WT and MDL-1^-/-^ mice after CLP induced sepsis. **(A)** IFN-γ, **(B)** IL-6, **(C)** TNF-α, **(D)** IL-10, **(E)** IP-10, **(F)** KC, **(G)** MCP-1, **(H)** MIG, **(I)** MIP-1. Data are depicted as the mean ± SEM of 3 independent experiments (*p < 0.05, **p < 0.01, ***p < 0.001, ****p < 0.0001, ns, no significance; two-way ANOVA).

To further unravel the immunomodulatory role of MDL-1 in sepsis-induced lung injury, we investigated the gene expression profiles of key proinflammatory cytokines and chemokines in lung tissues (**Figure 5**). This analysis aimed to provide deeper insights into the molecular mechanisms underlying the inflammatory response in the absence of MDL-1.

Our analysis revealed a significant upregulation of proinflammatory cytokine genes, including IFN-γ, IL-6, TNF-α, and IL-10, in the lung tissues of MDL-1^-/-^ -CLP mice compared to the WT-CLP group. This upregulation was particularly pronounced at the 24-hour mark post-CLP induction, highlighting the dynamic nature of the inflammatory response in the absence of MDL-1.

Notably, the gene expression of chemokine-related molecules, such as IP-10, KC, MCP-1, and MIP-1, was markedly elevated in the lungs of MDL-1^-/-^ -CLP mice compared to their WT-CLP counterparts. These chemokines play a critical role in recruiting immune cells to sites of inflammation, suggesting that MDL-1 deficiency may exacerbate immune cell infiltration and amplify tissue damage. The robust upregulation of proinflammatory cytokine and chemokine genes in MDL-1^-/-^ -CLP mice underscores the regulatory role of MDL-1 in tempering inflammatory pathways. The absence of MDL-1 appears to dysregulate the expression of these genes, leading to an uncontrolled amplification of the inflammatory response.

These findings collectively highlight the critical role of MDL-1 in modulating the expression of proinflammatory genes and chemokines during sepsis-induced lung injury. The observed dysregulation in MDL-1-deficient mice suggests that MDL-1 acts as a key checkpoint in controlling the inflammatory cascade. Targeting MDL-1 or its downstream pathways may offer a therapeutic strategy for mitigating excessive inflammation and improving outcomes in sepsis.

### 3.6 Impact of MDL-1 deficiency on the Syk/Caspase-3 pathway in CLP-induced sepsis

**Figure 6:**
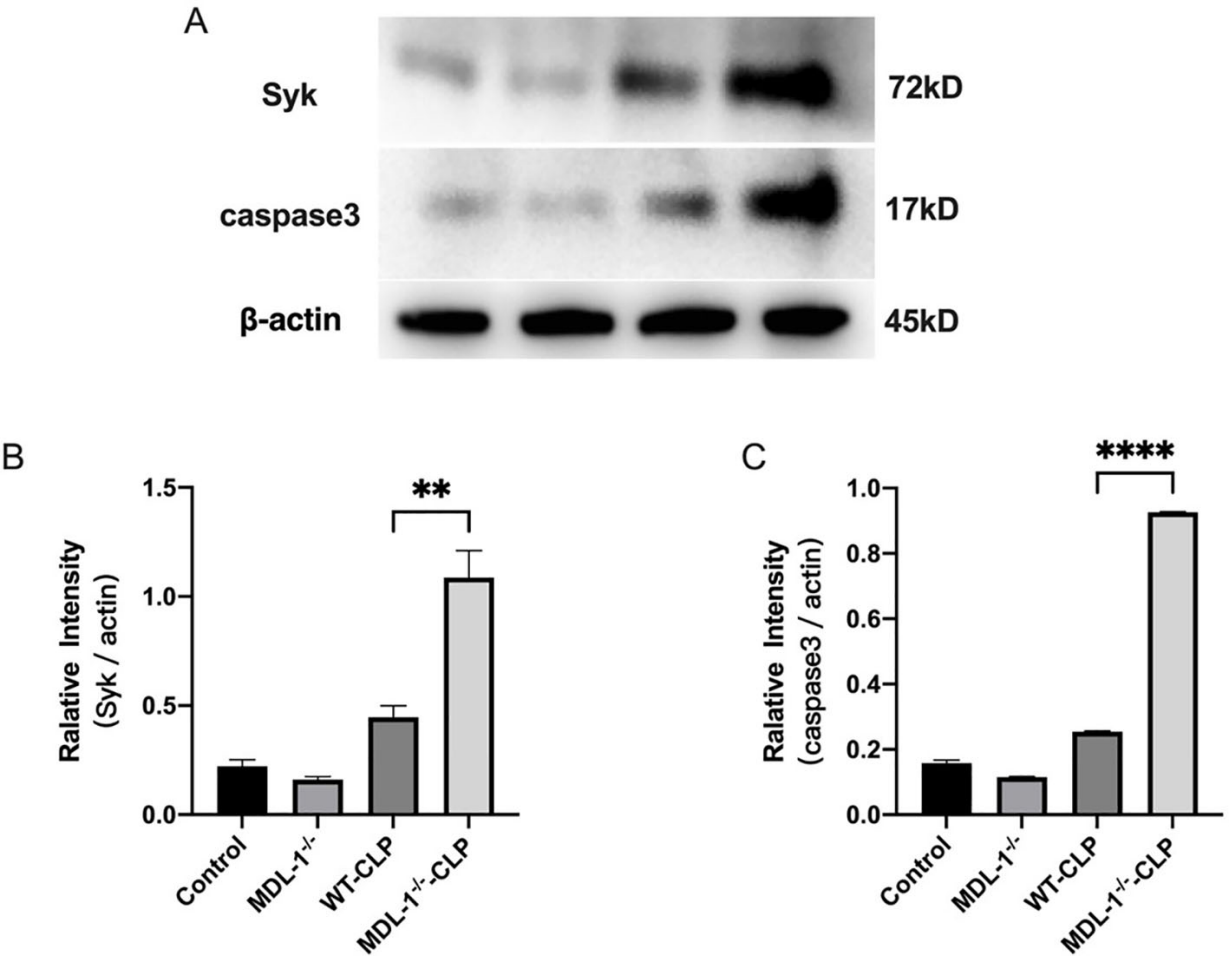
Western blot analysis and quantification of phospho-Syk (p-Syk) and cleaved caspase-3 in lung tissues of wild-type (WT) and MDL-1^-/-^ mice after cecal ligation and puncture (CLP). Western blot analysis of **(A)** phospho-Syk (p-Syk) and cleaved caspase-3 in lung tissues from WT and MDL-1^-/-^ mice post-CLP. **(B)** Quantification of p-Syk levels, **(C)** Quantification of cleaved caspase-3 levels. Data represent mean ± SEM (n=6-8/group; **p < 0.01, ****p < 0.0001), two-way ANOVA.

Apoptosis is a critical mechanism underlying the pathogenesis of lung injury in CLP induced sepsis. To explore the potential role of MDL-1 in regulating apoptotic processes, we measured the expression of Syk and cleaved caspase-3 in whole-lung tissue homogenates (**Figure 6A**).

Our analysis revealed a significant upregulation of Syk protein levels in the MDL-1^-/-^ -CLP group compared to the WT-CLP group (P < 0.01; **Figure 6B**). Additionally, we observed a marked increase in the expression of cleaved caspase-3 in the lung tissues of MDL-1^-/-^-CLP mice compared to the WT-CLP group (P<0.01; **Figure 6C**). These findings indicate that MDL-1 deficiency is associated with enhanced expression of Syk and cleaved caspase-3 in whole-lung tissue during CLP-induced sepsis. The findings also suggest that MDL-1 deficiency may lead to the activation of the Syk signaling pathway, which is known to play a role in promoting apoptosis. 

The intensification of both the Syk pathway and cleaved caspase-3 expression in MDL-1^-/-^ -CLP mice highlights the regulatory role of MDL-1 in modulating apoptosis. The absence of MDL-1 appears to dysregulate these pathways, leading to enhanced apoptotic cell death in lung tissues during sepsis. These findings provide novel insights into the mechanistic link between MDL-1 deficiency and the Syk and Caspase-3 pathway in sepsis-induced lung injury. The observed activation of apoptotic pathways in MDL-1-deficient mice underscores the protective role of MDL-1 in mitigating apoptosis and preserving tissue integrity. Targeting MDL-1 or its downstream signaling components may offer a therapeutic avenue for reducing apoptosis and improving outcomes in sepsis.

## 4 Discussion

Previous study had demonstrated MDL-1 gene was related to sepsis diagnosis and lung injury via screening multiple machine learning algorithms based on bioinformatics (10–12). This comprehensive investigation sheds light on the pivotal role of MDL-1 in the context of lung injury during sepsis (13). Our findings underscore the multifaceted contributions of MDL-1, not only in orchestrating the activation of the mononuclear phagocyte system and cytokine production but also in modulating the Syk-coupled pathway and apoptotic processes. Notably, our data reveal that MDL-1 deficiency renders mice more susceptible to CLP-induced sepsis, culminating in exacerbated lung inflammation and reduced survival rates.

The observed decrease in survival over a 7-day period in MDL-1^-/-^ mice aligns with previous observations in sepsis models (14) and glioma patients (15), further emphasizing the critical role of MDL-1 in septic conditions. The predominant expression of MDL-1 by myeloid cells, including macrophages and monocytes, is consistent with our findings in lung tissues and blood samples, suggesting its pivotal role in regulating the mononuclear phagocyte system and influencing lung injury dynamics. These observations are in line with studies that have highlighted the key role of MDL-1 in various inflammatory responses (16–18).

Previous studies have illustrated the inhibitory effects of blocking MDL-1 on inflammasome activation and cytokine production in response to viral infections (16). Our results further elucidate the intricate interplay of MDL-1 with various proinflammatory cytokines and chemokines, highlighting its potential involvement in orchestrating the immune response in septic lung injury.

These differential results could be attributed to the immune response to different types of pathogenic factors (14, 19). In our sepsis model, CLP-induced IFN-γ secretion occurs through MDL-1, whereas IL-6 and TNF-α secretion is mediated through other pathways. Additionally, MDL-1 deletion led to increased production of chemokines such as IP-10, KC, MCP-1, and MIP-1 following CLP, which in turn enhanced the immune response to sepsis. These findings suggest that the modulation of cytokine expression, along with increased chemokine production post-MDL-1 deletion, underscores the intricate balance of inflammatory pathways regulated by MDL-1.

The elevated levels of chemokines, such as IP-10, KC, MCP-1, and MIP-1, in the lungs of MDL-1^-/--^CLP mice further underscore the role of MDL-1 in influencing the balance of inflammatory pathways. The modulation of cytokine and chemokine expression highlights MDL-1’s critical role in orchestrating an effective immune response during sepsis. These findings are indicative of an exacerbated inflammatory milieu, which could contribute to the increased lung injury and decreased survival observed in MDL-1^-/-^ mice.

Notably, our study reveals a discrepancy between IFN-γ mRNA expression in lung tissue and protein levels in serum: while IFN-γ gene transcription is upregulated in MDL-1^-/-^-CLP lung tissue, circulating IFN-γ protein is reduced compared to WT-CLP mice. This mismatch aligns with previous reports that low serum IFN-γ levels during sepsis are associated with ‘immunoparalysis’, a state of impaired antimicrobial immunity that increases susceptibility to secondary infections (20). We speculate that MDL-1 deficiency may disrupt the systemic secretion or stability of IFN-γ, despite enhanced local gene expression in the lung. This dissociation between local and systemic IFN-γ responses underscores the complex immunomodulatory role of MDL-1 in sepsis, potentially regulating cytokine trafficking or tissue-specific cytokine bioavailability.

Insights into the interconnectivity between MDL-1 and signaling pathways involving p38, PI3K-Akt, and NALP3-inflammasome activation provide a deeper understanding of MDL-1-mediated immune responses (14, 21, 22). For example, dengue virus infection of dendritic cells results in the release of viral nucleic acids that interact with endosomal TLRs and cellular sensors to activate IRAK and TBK1 and induce the production of proinflammatory cytokines and interferons (23). Additionally, dengue virus activates MDL-1 via viral glycans to trigger NALP3-inflammasome formation and NFκB activation (24). Similar pathways are associated with H5N1 Influenza Virus infection (25). Previous study also revealed ovarian cancer proliferation could be mitigated through suppressing the MDL-1/PI3K-AKT feedback loop (26). We proposed that MDL-1 might be associated with apoptosis during sepsis. As MDL-1 is a Syk-coupled receptor, we examined the protein levels of Syk and caspase-3. Both Syk and cleaved caspase-3 were upregulated in the lungs of MDL-1^-/--^CLP mice. Our findings point towards a potential association between MDL-1 and the regulation of apoptosis, as evidenced by the upregulation of Syk and cleaved caspase-3 in MDL-1^-/--^CLP mice, further supporting the observed decrease in survival rates. This connection between MDL-1 and apoptosis regulation provides a novel insight into the underlying mechanisms driving lung injury during sepsis (27). However, the precise mechanisms governing this process warrant further investigation for a comprehensive delineation of the apoptotic pathways influenced by MDL-1 deficiency.

Regarding the observed upregulation of Syk and cleaved caspase-3 in MDL-1^-/-^-CLP lung tissue, we acknowledge several key limitations: (1) Whole-lung homogenates do not distinguish between cell types (e.g., immune cells vs. epithelial cells), so the cellular origin of elevated Syk and cleaved caspase-3 remains unclear; (2) downstream Syk signaling molecules were not evaluated, precluding confirmation of pathway activation; and (3) the relationship between Syk and caspase-3 is context-dependent—Syk has been shown to inhibit caspase-3 in certain cell types (28), suggesting that their co-upregulation may not reflect a direct causal link. Future studies using cell-specific knockout models or immunofluorescence co-localization will be necessary to clarify the cellular specificity and functional interactions between Syk and caspase-3 in MDL-1-mediated lung protection during sepsis.

Moreover, the potential therapeutic implications of these findings cannot be overstated. Targeting MDL-1 to modulate its activity could present a novel therapeutic strategy for mitigating lung injury during sepsis. The application of MDL-1 agonists or inhibitors could potentially balance the immune response, reduce excessive inflammation, and improve survival outcomes in septic patients (29). However, further research is warranted to explore these therapeutic potentials comprehensively.

While the study provides novel insights, certain limitations, such as the restricted scope of molecular investigations beyond Syk and cleaved caspase-3, underscore the need for additional research. Future endeavors focusing on MDL-1 agonists and a more expansive molecular analysis could further elucidate the intricate mechanisms underpinning MDL-1-mediated lung injury during sepsis. Future studies should also explore the cross-talk between MDL-1 and other pattern recognition receptors (PRRs) involved in the immune response to sepsis. Elucidating these interactions could provide insights into the broader regulatory networks influenced by MDL-1. Utilizing advanced techniques such as single-cell RNA sequencing could offer a more detailed view of the cellular and molecular changes occurring in the lungs during sepsis, particularly in the context of MDL-1 regulation.

In conclusion, our study underscores the critical role of MDL-1 in the pathogenesis of CLP-induced lung injury, with significant implications for immune modulation and survival outcomes in septic conditions. It is evident that MDL-1 deficiency increases the secretion of proinflammatory cytokines and chemokines, upregulates the expression of Syk and cleaved caspase-3, and thereby reduces the survival rate in a mouse model of sepsis. The potential therapeutic targeting of MDL-1 to mitigate pulmonary inflammation in sepsis holds promise. Further investigations to unravel the exact mechanisms and validate MDL-1 as a therapeutic target for sepsis-associated lung injury are warranted.

## Data Availability

The raw data supporting the conclusions of this article will be made available by the authors, without undue reservation.
